# Virus–Host Interaction Gets *Curiouser and Curiouser*. PART II: Functional Transcriptomics of the *E. coli* DksA-Deficient Cell upon Phage P1*vir* Infection

**DOI:** 10.3390/ijms22116159

**Published:** 2021-06-07

**Authors:** Grzegorz M. Cech, Agnieszka Szalewska-Pałasz, Katarzyna Potrykus, Anna Kloska

**Affiliations:** 1 Department of Bacterial Molecular Genetics, University of Gdańsk, Wita Stwosza 59, 80-308 Gdańsk, Poland; agnieszka.szalewska-palasz@ug.edu.pl (A.S.-P.); katarzyna.potrykus@ug.edu.pl (K.P.); 2 Department of Medical Biology and Genetics, University of Gdańsk, Wita Stwosza 59, 80-308 Gdańsk, Poland; anna.kloska@ug.edu.pl

**Keywords:** P1*vir* bacteriophage, DksA, RNA-seq analysis, phage development, host-virus interaction, transcriptomics

## Abstract

The virus–host interaction requires a complex interplay between the phage strategy of reprogramming the host machinery to produce and release progeny virions, and the host defense against infection. Using RNA sequencing, we investigated the phage–host interaction to resolve the phenomenon of improved lytic development of P1*vir* phage in a DksA-deficient *E. coli* host. Expression of the *ant1* and *kilA* P1*vir* genes in the wild-type host was the highest among all and most probably leads to phage virulence. Interestingly, in a DksA-deficient host, P1*vir* genes encoding lysozyme and holin are downregulated, while antiholins are upregulated. Gene expression of RepA, a protein necessary for replication initiating at the phage *oriR* region, is increased in the *dksA* mutant; this is also true for phage genes responsible for viral morphogenesis and architecture. Still, it seems that P1*vir* is taking control of the bacterial protein, sugar, and lipid metabolism in both, the wild type and *dksA^−^* hosts. Generally, bacterial hosts are reacting by activating their SOS response or upregulating the heat shock proteins. However, only DksA-deficient cells upregulate their sulfur metabolism and downregulate proteolysis upon P1*vir* infection. We conclude that P1*vir* development is enhanced in the *dksA* mutant due to several improvements, including replication and virion assembly, as well as a less efficient lysis.

## 1. Introduction

Infection of the cell by a virus triggers a sequence of events that may lead to the virus development, multiplication, and release from the host. The bacterial viruses, bacteriophages, serve as a safe model to study the virus-host interactions. The most studied bacteriophages include P1, lambda, and T4. The wide knowledge of the life strategies, genetics as well as its complete genome [1] placed the P1 phage among the best-known models of prokaryotic viruses. The P1 phage can infect many bacterial species from Enterobacteriaceae, however, the deepest knowledge of its development was obtained from the studies on *Escherichia coli*.

Upon infection, P1 chooses one of the two life cycles: lysis, leading to phage particle multiplication and propagation, or lysogeny when the P1 genome becomes an integral part of a bacterium in a form of a circular plasmid. The organization of the P1 genome, as is typical for viruses, strictly affects its functionality. The immunity regions: *imm*C, *imm*I, and *imm*T play a crucial role in the choice of the phage life strategy, as the main repressor, C1, and its inactivator, Coi are encoded there [2,3]. Moreover, an additional C1 anti-repressor function is achieved via the action of the Ant protein, whose expression is in turn regulated by C1 and other products of the *imm* region, namely the C4 antisense RNA and Icd [4,5]. The Icd protein also facilitates phage development by blocking bacterial cell division. The lytic cycle involves phage DNA replication initiated from the *oriL* region (while *oriR* serves as an origin to maintain lysogenic P1 plasmid replication) by the RepL initiatory protein. The host cell division is also blocked by the *kilA* gene product, encoded in the same operon with *repL* [6], ensuring that cellular resources are redirected to phage development. 

While the above-mentioned early genes are mainly responsible for regulatory processes and phage DNA replication, expression of the late genes is required for assembly of phage particles and subsequent host cell lysis; their expression is dependent on phage-encoded factor Lpa, which modulates transcriptional activity of the host RNA polymerase [7]. Phage lysozyme, controlled by a holin–antiholin system, ensures the timely and effective lysis of bacterial cells [1]. 

Transcription of the phage early genes is typically completed after 20 min of infection and the late genes are expressed without intermediate stages by 40 min, followed by cell lysis [1]. As is typical for intracellular parasites, P1 bacteriophage development depends on the host proteins in addition to phage-encoded regulatory and enzymatic apparatus. For example, P1 does not encode its own RNA polymerase [8]. Instead, it employs the host enzyme and directs bacterial transcription machinery, assisted by phage regulators (to express phage genes e.g., *c*1, *lpa*). In addition, the host-encoded SspA protein is required for transcription of the P1 late genes [9]. Thus, overall it can be assumed that modifications of host proteins involved in gene expression would directly affect bacteriophage development.

Among bacterial transcription factors that bind to RNA polymerase is DksA, which was first identified in *E. coli* as a multicopy suppressor of a temperature sensitivity phenotype due to mutations in chaperone-encoding genes, *dnaK* and *dnaJ* [10]. Some years later, this protein attracted more attention due to its function in transcription regulation. DksA was reported to act as a cofactor for the stringent response alarmone, (p)ppGpp, resulting in amplification of its negative or positive regulatory effects on transcription, depending on the specificity of a given promoter [11,12]. However, several lines of evidence indicate that DksA and (p)ppGpp actions could be independent or even antagonistic [13,14,15,16]. DksA is involved in the regulation of transcription initiating from σ^70^-dependent promoters, as well as in σ^54^-driven transcription [17]. DksA is defined as a global transcription regulator, interacting with the RNA polymerase via its secondary channel [18]. The role of DksA in some Gram-negative bacteria has been described and the presence of *dksA* homologs has been reported in many proteobacteria [19,20]. Mutants lacking *dksA* exhibit pleiotropic defects, e.g., in cell growth and division, stringent and stress response, quorum sensing, and DNA repair [19,21,22,23], indicating its important role in numerous cellular processes. A thorough transcription analysis aiming at DksA function was performed e.g., using microarrays to compare (p)ppGpp and DksA-deficiency in *E. coli* [14], transcriptomic analysis in *Pseudomonas aeruginosa* [24], *Borrelia burgdorferi* [25], or *Haemophilus ducreyi* [26]. 

The virus–host interaction requires a complex interplay between two opposite targets: phage strategy of reprogramming the host machinery to serve its purposes of propagation, and host cell defense against the infection [27]. Thus, at the molecular level, a complicated arrangement of gene expression alterations takes place, the process that can be studied employing various methods, e.g., computational approaches or Raman tweezers [28,29]. In this work, we attempted to resolve the phenomenon of improved lytic development of P1 phage in the DksA-deficient *E. coli* host by using a high-throughput approach to obtain a general view on the phage–host interaction. 

Here, we present the second part of our scientific story which is still getting *curiouser and curiouser*, which makes the reader amazed and brings to mind “*Alice’s Adventures in Wonderland*” by Lewis Carroll. We would like to encourage the reader to follow the beginning of this story in our accompanying paper: *Virus-Host Interaction Gets Curiouser and Curiouser. PART I: Phage P1vir Enhanced Development in an E. coli DksA-Deficient Cell* [30].

## 2. Results and Discussion

### 2.1. General Overview of the Virus–Host Transcriptome 

To obtain a widespread and holistic overview of the virus–host interactions, we analyzed the transcriptome of both, the P1*vir* phage and the *dksA* and wild-type hosts undergoing P1 infection. Total RNA sequencing was performed to assess the changes in gene expression pattern at 0, 10, and 30 min post P1*vir* infection. The choice of time points was based on the course of P1 lytic development—after 20 min, transcription of the early genes should be completed, the late genes are expressed up to 40 min, and then cell lysis occurs [1]. 

**Figure 1:**
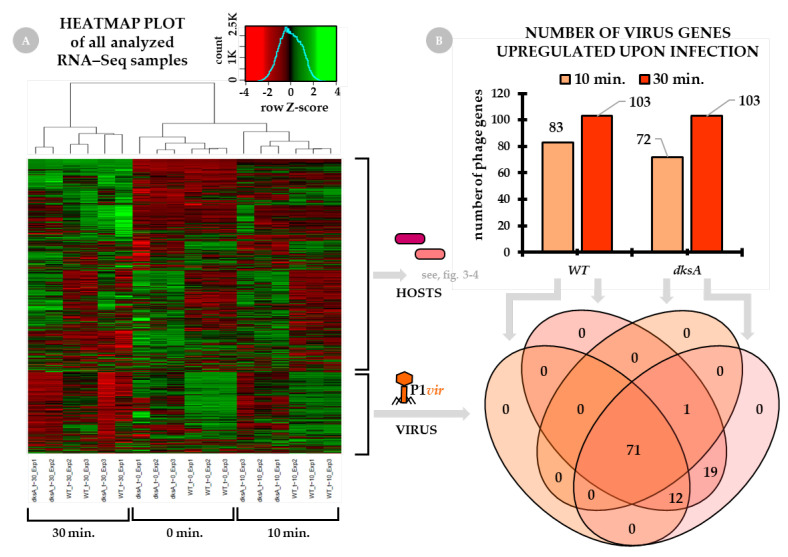
Changes in global gene expression in the course of the virus-host interactions. The wild type and the *dksA* mutant cultures were infected with the P1*vir* phage at the beginning of the logarithmic phase of growth. Total RNA sequencing (RNA-seq) was performed for three post-infection time-points: 0 min (no-phage control), 10 min (early gene expression), and 30 min (late gene expression). Panel (**A**) presents the heatmap plot of all analyzed RNA-seq samples based on the expression of all detected genes of both hosts (wild type and *dksA* mutant) and the P1*vir* virus. Sample replicates (three for each condition) are listed below the heatmap. Gene expression data are presented as Z-score transformed, scaled in rows values. Panel (**B**) shows the number of P1*vir* phage genes which have been expressed at 10 and 30 min of infection. The Venn diagram below panel B shows the number of P1*vir* genes upregulated in the wild type and *dksA* mutant cells.

The heatmap plot based on the expression of all detected genes of all sequenced samples is presented in Figure 1, panel A. As the sequenced samples for each time point and host were done in triplicate, a negative control was also included in the experiments–both hosts’ and the phage gene expression levels are well represented.

### 2.2. The Virus Transcriptome Upon Infection

*General overview of P1 phage gene expression.* Figure 1, panel B, shows the number of P1*vir* genes which have been expressed at 10 min and 30 min of infection. Roughly 70–80% of total phage genes are expressed at 10 min of infection. We found that at 30 min post-infection all phage genes are expressed. 

**Figure 2:**
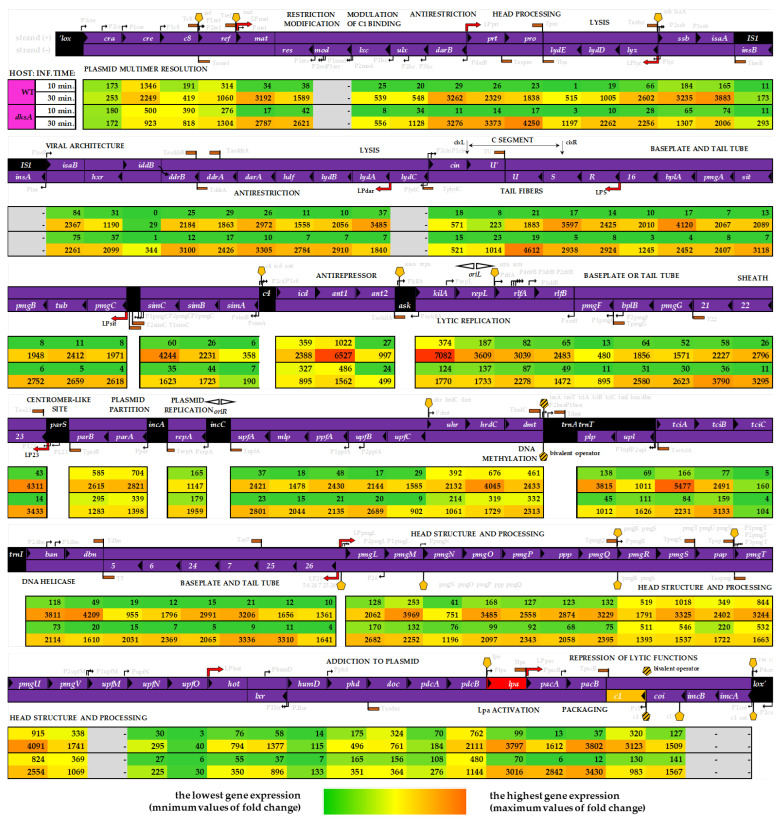
P1*vir* bacteriophage gene expression presented on the genome map. Purple ribbons indicate both coding strands (+/−); black triangles inside ribbons are pointing at a given gene’s transcription start site. P1*vir* transcriptome analysis upon infection is shown below the genome map. Gene expression fold changes are shown as values and heatmaps—the first two rows present data for the phage infecting the wild-type strain, and the next two rows present data for the phage infecting the *dksA* mutant; 10 min and 30 min post-infection, respectively. Several P1 genetic features are shown (gray description): σ^70^ promoters (black arrows); Lpa-dependent promoters (thick red arrows); C1 operator sites (yellow pentagon, patterned if C1 binds at dual sites) Rho-independent terminators are marked with brown flags; sequences other than protein-coding or intergenic regions are shown as black boxes; lytic and plasmid replication origins are shown as white arrows above the genome map. Depicted gene lengths are not proportional to their actual size.

In our analysis, we plotted the observed changes onto the P1*vir* genome map (Figure 2). This allowed linking the expression level of each gene at each time point and in each host with several known P1 genetic features, such as the σ^70^ promoters, Lpa-dependent promoters, C1 operator sites, or Rho-independent terminators. The level of gene expression differs between the time points and the hosts (Figure 2, see rows labeled WT and *dksA*). However, we did not find any uniquely expressed phage genes depending on the host. We also did not find an easy to define pattern in the hosts’ gene expression upon phage infection. It seems that a much more complex scenario is rolling out here. Even so, we are still able to pinpoint several factors or events that plausibly lead to the improved P1*vir* development in a DksA-deficient host (discussed below).

*The P1 virulence mechanism dilemma.* One of the most striking observations regarding the P1*vir* transcriptome in the wild type and *dksA* hosts is the difference in expression levels of phage genes from the C4-ant1/2 region (Figure 2). 

We found that the *ant1* gene which co-produces the lytic anti-repressor Ant is highly expressed in the wild-type host at 30 min post-infection. Moreover, the *kilA* gene, located directly downstream from the *ant1/2* genes is also highly expressed in the wild-type strain. These two genes show the highest level of expression among all of the phage’s genes during infection of the wild-type strain. 

High expression of the *ant1* gene can be linked to point mutations detected in the *imm*I region of the P1*vir* genome that we described in the accompanying paper [30]. This is because mutations located in the promoter region of the C4 antisense RNA or near the C4-antisense-RNA binding site may directly affect the *ant1* expression; thus, the permanent or extremely enhanced *ant1* expression forces P1*vir* to enter the lytic pathway. 

On the other hand, we must remember that in the wild type strain, although there is a much lesser expression of *ant1* and *kilA* genes, those genes are still highly expressed in compering to the general gene expression in the wild type host, (Figure 2, both hosts in 30 min). As mentioned in the accompanying paper [30], our findings support the hypothesis that originated in the 1980s claiming that the mutation located somewhere within the C4-ant1/2 region is a key to the P1*vir* obligatory virulence [31]. 

The KilA protein promotes cellular death, so very high levels of *kilA* gene expression will promote lytic development. Moreover, KilA has been considered as another anti-repressor of the lytic functions (prof. M. Łobocka, personal communication). This hypothesis is even more interesting if we consider the expression of the P1*vir ant1/2* and *kilA* genes in the *dksA^−^* host. Expression of these two genes in the *dksA^−^* host is also sufficiently high for the P1*vir* phage to enter the lytic development, but the overall expression level of these genes is much lower in comparison to the one in the wild type host. We hypothesize that lower expression of genes directly responsible for triggering the lytic-only development may result in a less rapid and more gentle lysis.

*The gentle lysis hypothesis.* We hypothesize that the lack of DksA protein unlocks a cascade of events which leads to a situation where cell lysis is less efficient and somehow less rapid, thus, maturation of more virion particles may be possible. As we have shown in both, the qPCR (in the accompanying paper [30]) and RNA-seq analysis (this work), the level of phage *lpa* mRNA decreases more significantly in the DksA-deficient strain. The Lpa protein activates the expression of the so-called Lpa-controlled operons and is required for expression of the P1 late genes, e.g., the lysozyme, structural proteins, and DNA packaging proteins. With some exceptions, genes involved in the late functions are expressed from late promotors and are entirely or partially dependent on Lpa [1]. The level of *lyz*-specific mRNA is lower when the phage infects the *dksA* mutant than in the wild-type bacteria. Moreover, we have observed upregulation of the phage *lydE* and *lydB* gene expression in the *dksA* host (Figure 2); these genes encode antiholins which are lysis determinants that prevent premature lysis [1]. In contrast, we observed that expression of the phage *lydA* gene encoding holin, a protein triggering cell lysis, as well as the *kilA* gene, a protein whose overexpression kills the host cell [32], are substantially downregulated in the *dksA* mutant in comparison to the wild type host, at 30 min post-infection (Figure 2). We think that the less efficient expression of genes responsible for the host cell lysis may give a chance for more efficient phage DNA replication and virion part assembly before all of cell barriers collapse when cell lysis occurs and progeny virions are released. This potential shift could explain the improved lytic phage development observed in the *dksA^−^* strain—the longer the bacterial cells are kept in a good condition and the antiholins prevent premature lysis, the more virus particles will be able to mature in a given time. Additionally, RNA-seq analysis proves experimental observation of lower *c*1 gene expression under given conditions (the accompanying paper [30]).

*P1vir in a dksA^−^ strain—at the brink of lysogeny.* Despite the lysis-only development of P1*vir*, our analysis of phage transcriptome revealed some changes in the expression of genes involved in the lysogenic pathway upon infection of the *dksA* strain. It was claimed previously that the P1*vir* phage is unable to enter the lysogenic cycle due to a mutation located in the immunity region *imm*I [31]. We show that in the *dksA* strain expression of the *ant1* and *ant2* genes located in this region is downregulated at both post-infection timepoints (Figure 2). Moreover, *icd*, another gene of the *imm*I region, is downregulated in the *dksA* strain at 30 min post-infection (Figure 2); expression of this gene causes reversible inhibition of cell division and apparently is required for *ant* expression [1,33,34]. Intriguingly, the *c*8 gene is upregulated in the *dksA* strain at both time points of infection. Previously published data suggested that the *c*8 gene is probably involved in the establishment of lysogeny [35,36], thus, its upregulation may be difficult to explain because P1*vir* can solely undergo lytic development. We speculate that the enhanced expression of the lysogeny-related genes even in the lytic-only P1*vir* phage is leading to a higher phage yield in the *dksA* strain. 

*Minor improvements in the phage development.* Although very attractive, gentle lysis of the host may not be enough on its own to allow the production of more virion particles. Literally, phages need more parts to build up the virions. First, more copies of phage genomes would be needed to produce more progeny phages. Indeed, we observed an enhanced replication of the mini-P1 replicon in the *dksA* mutant strain (see accompanying paper [30]). Additionally, transcriptome analysis revealed that the *repA* gene expression is increased in the *dksA* mutant when compared to the wild-type strain. RepA is necessary for DNA replication initiating at the phage *oriR* region and it is needed at the early stages of P1 development, as well as during lysogeny. P1 also has another replication origin—*oriL*, but the expression of *repL*, a gene encoding the initiator protein of *oriL*-driven replication, is not enhanced in the *dksA^−^* strain when comparing to the wild type cells (Figure 2). Phage DNA from both of these origins is replicating by the theta model, however, in the late stages of lytic development replication switches to the sigma model. It should not be assumed that one origin (*oriR*) is strictly lysogenic and the other (*oriL*) is strictly lytic; again, the regulation of P1 replication is complex and shows some flexibility [1]. Nevertheless, we can assume that stimulation of replication from at least one origin will lead to a higher copy number of phage genomes that can be used to assemble progeny virions in the *dksA* host.

Upregulation of genes that are involved in viral particle formation could also explain the improved phage lytic development observed in the *dksA^−^* strain. To produce more phages, more virion parts would be needed. Indeed, we noticed, that several morphogenesis and viral architecture genes are expressed during phage development. In the DksA-deficient strain, 30 min post-infection, expression of several phage genes whose functions are linked to capsid development is substantially upregulated (Figure 2); these include the following genes: *5*—encodes the baseplate; *6*—encodes tail length determinant; *25*—encodes tail stabilizing protein [37]; *prt*—encodes portal protein; *pro*—encodes putative head processing protease and kinase and is required for head morphogenesis [1]; *ddrA*—encodes viral architecture determinant but its full function is uncertain [38]; *U*—encodes the virion protein *gpU*′ when expressed in (+) orientation; *U′*—variable gene of tail fiber operon, encodes tail fiber assembly chaperone when expressed in (−) orientation [1]; *R*—tail fiber structure or assembly [39]. 

Overall, the transcriptome analysis of P1*vir* infection shows that changes in phage gene expression result from a complex interplay between the phage and the host. This situation could be explained in the light of P1 phage biology. This phage can develop in many Enterobacteriaceae hosts, e.g., *Shigella dysenteriae*, *Salmonella typhimurium, Klebsiella pneumoniae,* or *Enterobacter aerogenes*. It can also adsorb and inject its genetic material into other Gram-negative bacteria such as *Agrobacterium tumefaciens, Alcaligenes faecalis*, or *Myxococcus xanthus* [40]. Thus, the control of phage development should be multi-level and highly adaptable. Several of the Lpa-controlled genes have additional σ^70^ promotors—this may suggest multiple ways to regulate them, possibly allowing adaptation to alternative hosts or environmental conditions [1]. Indeed, not all genes of the Lpa-controlled operons are associated with the expression level of the *lpa* gene. Moreover, in the *dksA* strain, there are at least several upregulated phage genes, e.g., *upl*, *tciB*, *pmgN*, and *iddB* (Figure 2) of unknown function [1].

*Summary of this section.* Upon P1*vir* phage infection, most of the phage genes are expressed in both tested hosts, the wild-type strain and the *dksA* mutant. It corresponds to the mechanistic nature of viruses—if the host is fully suitable, the development is completed. Compared to their hosts, viruses have minimalist genomes, therefore phage genes often encode proteins with multiple functions. This may be the reason why we did not observe any “general” genetic switch that would be suppressed in the presence of DksA and activated in its absence. The whole phage transcriptome appears to be modulated in a much more subtle way. Analysis of phage gene expression levels upon infection of the wild type and DksA-deficient hosts enabled us to find some transcription patterns explaining better development of P1*vir* in the *dksA* strain. On one hand, we found a much higher expression of P1*vir* genes of the *imm*I region in the wild-type host, most probably leading to the virulence of this phage. On the other hand, a lower level of expression of these genes makes the beginning of lysis less efficient in the *dksA* mutant host. We propose a "gentle" lysis hypothesis where we claim that the less efficient rupturing of the host cell is one of the key contributors to a more enhanced development in the DksA-deficient host. It seems that when developing in the *dksA* host, P1*vir* is at the brink of lysogeny but is unable to establish it and eventually enters the lytic pathway. In this scenario, several minor improvements in phage development are also essential, such as stimulated DNA replication or enhanced production of virion parts. However, without further research, it is still difficult to figure out every mechanism of a possible direct and indirect role that DksA plays in phage gene expression and phage development. Nevertheless, we are proposing some.

### 2.3. The Host Transcriptome Upon Infection

Host transcriptomic data for the wild type and *dksA* strains upon P1*vir* infection at 0, 10, and 30 min time points were obtained from the same experiment as described above.

**Figure 3:**
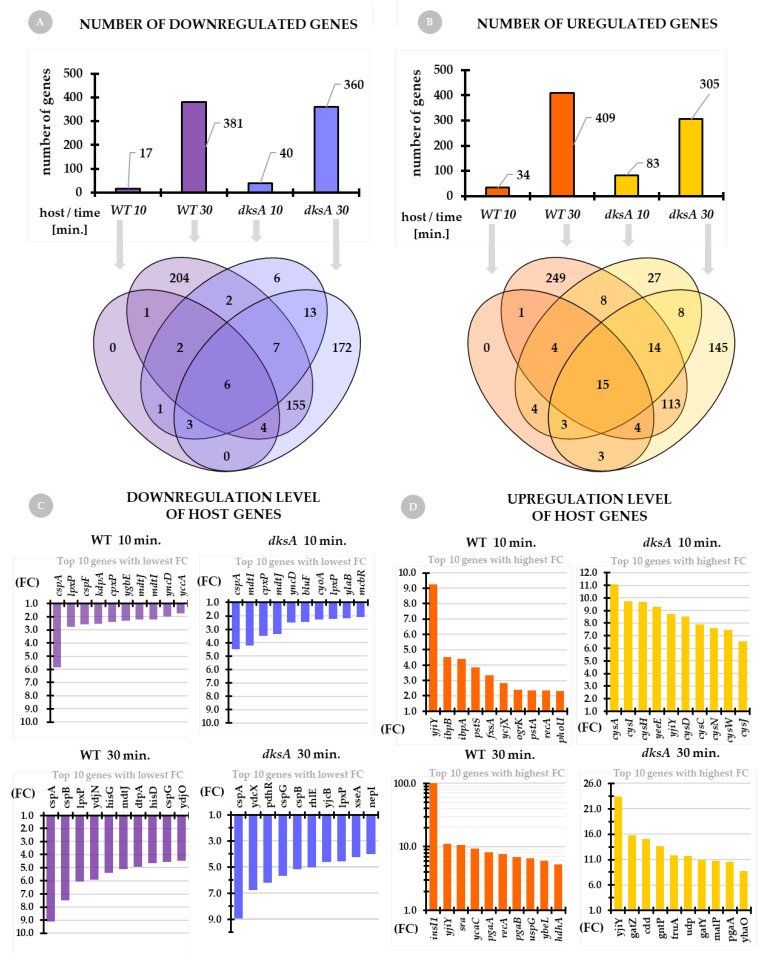
General overview of the host transcriptome upon P1*vir* infection. Panel (**A**) shows the number of downregulated genes of both, the wild type and *dksA* mutant hosts at 10 min and 30 min post-P1*vir* infection. Panel (**B**) shows the number of upregulated genes of both hosts at selected time points upon P1*vir* infection. Venn diagrams below panels A and B show the distribution of up- or downregulated genes. Panel (**C**) shows the downregulation level of the top ten genes with the lowest expression at each time-point and host. Panel (**D**) shows the upregulation level of the top ten genes with the highest expression at each time-point and host. Genes with Fold Change (FC) > 1.5 were selected for further analysis. Gene descriptions are in the text and Appendix A, Table A1.

*General overview of the host transcriptome*. The number of downregulated genes of the wild type and *dksA* mutant strains is presented in Figure 3A. For both host strains, at 10 min of infection, we see a much smaller pool of downregulated genes in comparison to 30 min of infection. A slightly higher number of downregulated genes is noted for the wild-type strain (381) when comparing to the *dksA* strain (360) at 30 min post-infection. On the other hand, at 10 min the number of downregulated host genes of the *dksA* mutant is twice that of the wild-type strain. Analysis of the distribution of these genes shows that certain gene sets overlap between the hosts and time points, while others are uniquely downregulated solely in the wild type strain or solely in the *dksA* mutant; this mainly concerns the genes expressed at 30 min post-infection (Figure 3A, Venn diagram).

We observe a similar situation concerning the number of host genes that are upregulated upon phage infection (Figure 3B). For both tested host strains, at 10 min of infection, we see a much smaller number of upregulated genes when compared to 30 min post-infection. A higher number of upregulated genes appears in the wild type strain (409) when compared to the *dksA* host (305) at 30 min of infection, however, much more genes are upregulated in the *dksA* mutant (83) at the 10 min time point than in the wild type strain (34). Analysis of the distribution of upregulated genes between the hosts and time points shows that some upregulated genes overlap, but others are uniquely upregulated in the wild type of strain (mostly at 30 min) or the *dksA* mutant (at both, 10 min and 30 min of infection) (Figure 3B, Venn diagram). 

Since the number of genes (in both hosts) that are up- or downregulated is very large, it is impossible to present them here one-by-one, and thus we decided to discuss here only the top ten genes with the lowest expression level (downregulated) and the top ten genes with the highest expression level (upregulated) for each host and each time-point (Figure 3C,D, respectively). For further details, see Supplementary Materials of the accompanying paper [30].

Interestingly, we found that the *cspA* gene encoding the cold shock protein A (CspA) was the most downregulated gene at each time-point and each host. CspA is the major cold-shock protein whose mRNA is very unstable at 37 °C and its levels rapidly decrease during growth; its expression levels change in response to temperature fluctuations [41,42,43]. It was shown that *cspA* is expressed only during the early-log-phase of growth at 37 °C and during the log phase after a shift from 37 °C to 10 °C [44,45]. However, *cspA* is also expressed at 37 °C under nutritional up-shift conditions, before the cells start to divide [46]. Downregulation of this gene’s expression upon P1*vir* phage infection is a new and interesting discovery raising questions of its role in the process of P1*vir* taking over the host metabolism [47]. Indeed we did find several cold shock protein-encoding genes to be downregulated (Figure 3C and Appendix A, Table A1). Response to cold shock is a kind of a global reprogramming hub allowing bacterial cells to adapt. Turning down this pathway in the cells seems to provide a metabolic profit for the virus–cold shock regulatory features became a very useful and versatile tool for taking control of the host. The *cspA* gene expression is also shown to be induced by the addition of chloramphenicol [48,49]. Moreover, genes encoding multidrug efflux system transporters, such as *mdtI* and *mdtJ*, are downregulated upon infection (Figure 3C and Appendix A, Table A1). We hypothesize that besides preserving energy and substrates, one of the P1*vir* phage strategies is to alter systems involved in cellular responsiveness to environmental threats. Using global regulators as multipurpose tools seems to be an effective way for taking control of hosts, also dampening the responsiveness of cellular alert systems. 

We found that the *yjiY* gene (synonym: *btsT*) is the most upregulated gene at almost each time point and in each host (Figure 3D). This gene encodes a high-affinity, pyruvate/H^+^ symporter which mediates the uptake of pyruvate under nutrient limiting conditions [50]. Expression of *btsT* is induced at the onset of the stationary phase in media containing peptides or amino acids as the source of carbon [51,52]. Interestingly, *btsT*, among other genes involved in carbon source transport and metabolism, was downregulated in two MG1655 lysogens carrying closely related Stx2a phages—O104 and PA8 [53]. It seems that P1*vir* has an opposite strategy and upregulates this symporter as an energy/carbon source provider. In the wild-type strain, at 30 min of infection, we found *insI1* as one of the most upregulated genes. In contrast, this gene was not present in any set of genes upregulated upon infection in the *dksA* host. The *insI1* gene encodes a transposase for the IS30 insertion sequence. This transposase interacts with the terminal inverted repeats of the IS30 sequence element, using them as targets for transposition [54,55]. Why DksA-deprived cells are unable to express this gene remains unclear. We speculate that in the wild-type cells, phage infection stimulates the excision of this mobile genetic element. Cell lysis, as a result of phage infection, may be a mechanism of propagation of the IS30 element in the bacterial population. The lack of DksA, a major global transcriptional regulator, abolishes this process. 

We found an even more interesting situation in the *dksA* strain at 10 min post-infection. The pool of the top ten most highly expressed genes is represented mostly by genes involved in the sulfur metabolism—*cysA*, *cysI*, *cysH*, *cysD*, *cysC*, *cysN*, *cysJ*, *cysW* (Figure 3D and Appendix A, Table A1). These genes are not expressed either at 30 min of infection in the *dksA* mutant or the wild-type cells at any time point. Sulfate assimilation proceeds by consecutive steps of its import (carried out by a protein encoded by *cysA* [56,57]), adenylation (encoded by *cysD, cysN*), and phosphorylation (encoded by *cysC*), prior to sulfate reduction to sulfite coupled to its release (encoded by *cysH*). CysH encodes a 3’-phospho-adenylylsulfate reductase that releases sulfite and adenosine-3,5-bisphosphate (PAP). The *cysI* encodes a subunit of sulfite reductase which is involved in the assimilation of sulfate and catalyzes the electron transfer from NADPH to sulfite to produce sulfide [58]. Moreover, *cysD* belongs to a network of genes that facilitate stress-induced mutagenesis (SIM) in *E. coli* K-12 [59]. Interestingly, *cysD*, as well as other genes involved in the sulfur metabolism, were also significantly upregulated in the MG1655 lysogen carrying the Stx2a phage PA8 [53].

**Figure 4:**
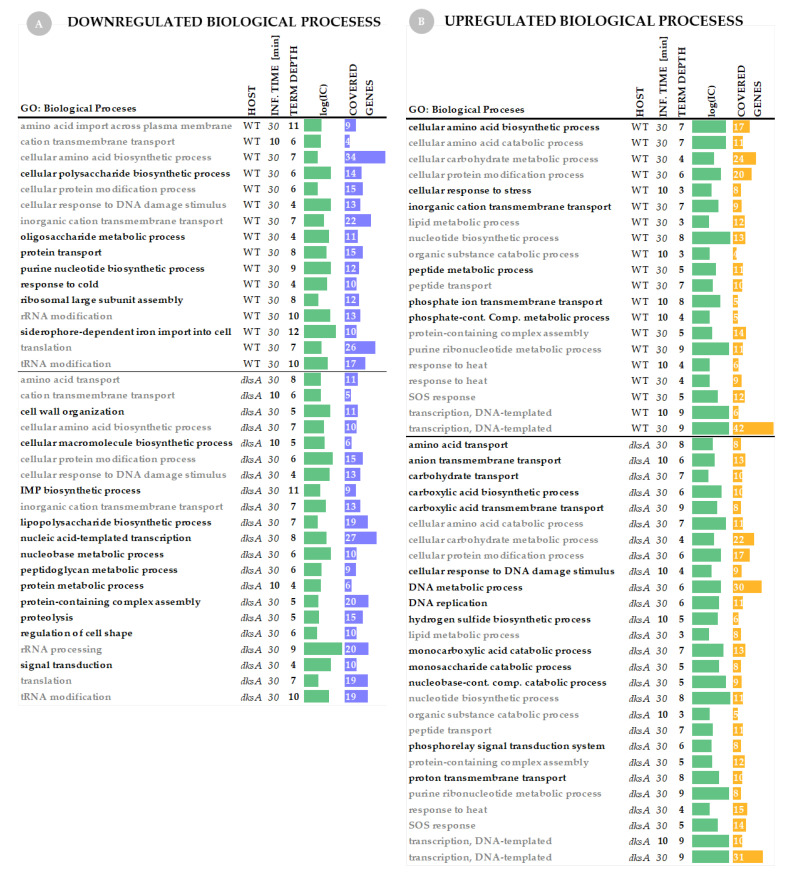
Gene Ontology (GO) terms analysis of the host genes upon P1*vir* infection. Panel (**A**) shows downregulated biological processes in the wild-type strain and the *dksA* mutant. Panel (**B**) shows upregulated biological processes in both tested hosts. The horizontal line indicates a border between the host sets of GO terms. Descriptions in gray represent GO terms that are common to both hosts. Descriptions in black represent biological processes that are uniquely up- or downregulated in a given host. We used the GSAn algorithm exploiting the semantic of concepts in Gene Ontology which provided a reduced and synthetic number of GO terms resuming the biological role of the gene set input. The Information Content (IC) is a score associated with the terms explaining how much they are informative (the bigger the IC is, the more specific the term is); log(IC) is used for visualization purposes. Term depth (number of GO terms united together after semantic analysis) and covered genes (the number of genes whose expression has been up- or downregulated) are also provided.

*Gene Set Annotation—analysis of Gene Ontology terms.* Since it is impossible to analyze all genes one by one in each gene set, we performed a Gene Set Annotation which provided summarized information about biological processes that were down- and upregulated at selected time-points and hosts upon P1*vir* phage infection (Figure 4).

*Biological processes regulated in hosts upon phage infection.* We found that the host gene expression changes (both, up- and downregulated pool of genes) are more pronounced at 30 min of infection in comparison to 10 min (Figure 3), and this is also reflected in Gene Ontology terms analysis. In the wild-type strain, only one biological process—the cation transmembrane transport—is downregulated at 10 min post-infection (Figure 4A). In the *dksA^−^* strain we have also found only a small set of downregulated biological processes (Figure 4A). Among upregulated biological processes at 10 min of infection, we found six GO terms for the wild type and five for the *dksA* strain (Figure 4B). Changes occur in more biological processes (up- and downregulated) at 30 min of infection—this applies to both, the wild type and the *dksA* mutant strains (Figure 4A, B).

*Downregulated biological processes in the host cell.* Based on the GO term analysis, we found many similarities in the downregulation of metabolic processes between both hosts upon P1*vir* infection. In both hosts, we found downregulation of amino acid import across the plasma membrane, cation transmembrane transport, cellular amino acid biosynthetic process, cellular protein modification process, cellular response to DNA damage stimulus, rRNA modification (Figure 4A). The first response to phage infection is shutting down biosynthesis of macromolecules (including proteins) and some transmembrane transporters—it appears that the energy-consuming systems are downregulated and host metabolism starts to be reprogrammed for phage purposes. That kind of strategy is general for many viruses, including phages, however, it may differ in details and execution [1,53,60]. On the other hand, another important phage strategy upon infection is the downregulation of cellular response to DNA damage stimulus which we found in both hosts (Figure 4A)—this may result from phage DNA maintenance and protection. Those processes also fit well with the phage proceeding to take over cellular metabolism [1,6,53].

*In the wild type.* Unique to the wild-type host, we observed downregulation of siderophore-dependent iron import into the cell, cellular polysaccharide biosynthesis process, purine nucleotide biosynthesis process, response to cold, and ribosomal large subunit assembly (Figure 4). In general, we can observe more detailed scenarios for energy conserving processes, such as reduced synthesis of poly- and oligosaccharides, complex compounds or their modifications, assembly, or de novo synthesis of basic compounds.

*In the dksA^−^ strain*. Unique to the *dksA* host, we observed downregulation of the peptidoglycan metabolic process, inosine monophosphate (IMP) biosynthetic process, lipopolysaccharide biosynthetic process, signal transduction, nucleobase metabolic process, cell wall organization and regulation of cell shape, and proteolysis (Figure 4A). We see that here the scenario of conserving energy and resources is a little bit different in execution than in the wild-type host. Downregulation of proteolysis in the *dksA* mutant cells infected with P1*vir* is particularly interesting, as two other bacteriophages—λ and T4—have been also shown to affect proteolysis in an infected cell by acting on the host-encoded proteases (reviewed in [27]). However the phenomenon of increased phage T4 yield in a *dksA^−^* mutant was described only partially—the host transcription was not performed in the analysis [61]. In addition, the fact that we observed the inhibition of expression of genes involved in the by P1*vir* only in the *dksA^−^* host supports our gentle lysis hypothesis—less efficient protein degradation in the host cell may support more enhanced phage development.

*Upregulated biological processes in the host cell*. Just like for the downregulated processes, we found many similarities in the upregulation of metabolic processes between both hosts upon P1*vir* infection. In both hosts, we found upregulation of the organic substance catabolic process, cellular amino acid catabolic process, cellular protein modification process, peptide transport, protein-containing complex assembly, cellular carbohydrate metabolic process, lipid metabolic process, nucleotide biosynthetic process, purine ribonucleotide metabolic process, transcription (DNA-templated), response to heat, and the SOS response (Figure 4B). In general, phage P1*vir* controls the hosts by upregulating their protein, sugar, and lipid metabolism, while the hosts try to take action by activating the SOS response or upregulating their response to heat.

Unique to the wild-type strain, we observed upregulation of inorganic cation transmembrane transport, phosphate ion transmembrane transport, phosphate-containing compound metabolic process, cellular amino acid biosynthetic process, peptide metabolic process, and general cellular response to stress (Figure 4B). 

Unique to the *dksA* strain, we observed upregulation of amino acid transport, anion transmembrane transport, carbohydrate transport, carboxylic acid transmembrane transport, proton transmembrane transport. Besides stimulation of the resources-gaining processes, we also observed upregulation of the monocarboxylic acid catabolic process, monosaccharide catabolic process, nucleobase-containing compound catabolic process, carboxylic acid biosynthetic process, and phosphorelay signal transduction system (Figure 4B). Moreover, upon P1 infection *dksA* cells promote the DNA metabolic process, cellular response to DNA damage stimulus, and DNA replication (Figure 4B). 

Indeed, we observed stimulated DNA replication of the mini-P1 replicon in the *dksA* mutant (see accompanying paper [30]). We have found that expression of the *repA* gene, the phage *oriR* replication initiation protein, is increased in the *dksA* mutant when compared to the wild-type strain [30]. Thus, if DNA replication is stimulated in the *dksA* mutant, more phages can be produced.

Even more interesting and striking is the upregulation of the hydrogen sulfide biosynthetic process that we found unique to the *dksA* host (Figure 4B). This biological process appears in Gene Ontology analysis as a result of the upregulation of many sulfur-metabolism-linked genes, which were discussed in a previous section. Many prokaryotic species generate hydrogen sulfide, but the biochemistry and physiological role of this gas in non-sulfur utilizing bacteria remain largely unknown. However, the inactivation of key enzymes in sulfur metabolism in *Staphylococcus aureus, Bacillus anthracis, Pseudomonas aeruginosa*, and *Escherichia coli* suppresses H_2_S production makes certain pathogens highly sensitive to a multitude of antibiotics [62]. The mechanism of H_2_S-mediated antibiotic resistance relies on the modification of oxidative stress imposed by antibiotics [62]. Is activation of sulfur metabolism a universal mechanism of preparing the cell to cope with environmental threats? We have previously reported that the H_2_S metabolism is activated in the marine bacterium *Shewanella baltica* upon cold stress [63]. We suggested there that the cold stress may activate some metabolic preparation pathways for upcoming anaerobic conditions. In addition, a study by Berger et al. revealed a profound impact of the Stx phage presence in *E. coli* on carbon source utilization and sulfur metabolism [53]. The Stx2a prophage appears to reprogram the carbon metabolism of its bacterial host by turning down aerobic metabolism in favor of mixed acid fermentation [53]. It was shown that many sulfur metabolism pathways were upregulated [53]. Here, we also observed such changes and thus hypothesize that the upregulation of sulfur metabolism may be a general, non-specific cellular response to various environmental threats.

*Summary of this section.* After analysis of the hosts’ transcriptomes upon P1*vir* infection, we found that intensive cellular reprogramming is held mostly at 30 min of infection. However, we also observed some important events unique to the *dksA* mutant at 10 min of infection, such as the upregulated expression of genes directly involved in the sulfur metabolism. How exactly P1*vir* development benefits from this upregulation remains unclear, however, we speculate that this may be linked to obtaining new energy sources or global reprogramming via H_2_S signaling functions. Moreover, we have discovered downregulation of cold-shock genes and upregulation of heat shock genes. Since cold shock and heat shock proteins usually play multiple roles in bacterial cells, by employing globally-acting factors, the phage can efficiently take over the host metabolism. The shifting expression of global-acting regulators may be an elegant element of the control-taking strategy.

By analyzing Gene Ontology terms, we can see how the host cells are reprogramed by P1*vir* and how this reprogramming is reflected in cellular metabolism. In general, there are many similarities between metabolic responses of both hosts to phage infection. Phage P1*vir* is taking control of the protein, sugar, lipid, and nucleotide metabolism. We observed downregulation of nonessential and energy-consuming bacterial processes, e.g., some biosynthetic pathways, and transport or response to DNA damage stimulus, and upregulation of processes essential for phage development, e.g., several catabolic processes or nucleotide biosynthesis. Both hosts are reacting by activating the SOS response or upregulating the heat shock proteins. 

We can also find interesting differences between the hosts in metabolic response to phage infection, which may reflect slightly different ways in which the phage executes its strategy for obtaining energy and resources during development. Upon infection, the *dksA* host upregulates transport of different molecules and several catabolic processes, including hydrogen sulfide biosynthesis, as well as the DNA replication process. Moreover, downregulated proteolysis in the *dksA* host in the later stage of infection may also influence the regulation of phage development. All of these observations fit into the "minor improvements" scenario that results in the enhanced development of P1*vir* in a DksA-deficient host when combined.

## 3. Materials and Methods

### 3.1. Bacterial Strains and Phages

Bacterial strains used in this work: CF1648—MG1655 wild type [64]; CF9240—MG1655 *dksA*::Tn10 [23]. Cultures were routinely grown at 37 °C in lysogeny broth (LB) supplemented with antibiotics as needed (tetracycline 15 µg/mL). Phage P1*vir* from the collection of the Department of Bacterial Molecular Genetics, University of Gdańsk, Poland.

### 3.2. Total RNA Isolation

Bacterial wild-type and *dksA* strains were cultured in LB broth containing 10 mM CaCl_2_ to OD_600_ = 0.2, at 37 °C with shaking. Next, P1*vir* was added at final MOI = 10. One-ml samples were immediately withdrawn to previously prepared 1.5 mL tubes containing 250 µL of aqua-phenol (5% phenol in 96% ethanol). Samples were withdrawn at 0, 10, and 30 min after P1*vir* infection. Next, RNA isolation was performed using the RNeasy Mini Kit (Qiagen, Hilden, Germany) according to the manufacturer’s protocol. The elution step was performed twice using 50 µL of RNase-free water on the same column to increase the RNA yields. Thus obtained RNA was digested with a DNase using TURBO DNA-free™ Kit (Ambion, Carlsbad, CA, USA), according to the manufacturer’s protocol. Samples were stored at −80 °C. The purity and integrity of RNA were assessed using the Bioanalyzer 2100 (Agilent Technologies, Santa Clara, CA, USA) and Agilent RNA 6000 Nano Kit, according to the manufacturer’s protocol.

### 3.3. RNA-Seq Analysis

Total RNA was isolated and evaluated for quality by using the Bioanalyzer 2100 as described above. The sequencing run was conducted on the Illumina NovaSeq6000 platform (Macrogen Inc., Seoul, Korea). The library was prepared using the following kit: Ribo-Zero rRNA Removal Kit (Bacteria), TruSeq RNA Sample Prep Kit v2 (Macrogen Inc., Seoul, Korea) according to the following protocols: Ribo-Zero User Guide, TruSeq RNA sample prep v2 Guide, Part # 15026495 Rev. F. 

### 3.4. High Throughput Data Analysis

30 million pair-end reads per sample were assessed with 101 bp read length. Reference P1 phage genome sequence (NC_005856.1) and annotations were downloaded from GenBank. Quality and adapter trimming of the short reads was performed using Trimmomatic [65]. Short reads matching known rRNA sequences were removed using the HISAT2 aligner [66]. Read quality reports before and after quality filtering were prepared using the FastQC software v0.11.7 [67]. Filtered reads were aligned to the reference genome using Burrows-Wheeler Aligner with the selected BWA-MEM algorithm [68]. The Sambamba software was used for BAM file processing [69]. Read mapping reports were created using the Qualimap software [70]. RSEM (RNA-Seq by Expectation Maximization) [71] was used to quantify the expression values of genes. Additionally, Salmon [72] was used to quantify the expression values of genes (not used in further analysis). Hierarchical clustering of RNA-seq samples (Pearson correlation metric, centroid linkage) based on the expression values of all genes was performed using standard R functions (R Core) and variance stabilizing transformation was provided by the DESeq2 package [73]. Differential expression analysis between designated groups of samples was performed using the voom+limma pipeline [74]. The false discovery rate (FDR) threshold of 0.01 and a fold change threshold of 1.5 were used in the analysis. Gene Set Annotation (GSA) was done using GSAn 1.0.5, a public web server for characterizing gene lists of high-throughput genomics [75]. Glimma package [76] was used to provide interactive graphics—Interactive HTML Volcano plots, Interactive HTML MA plots (Suplementary_plots_Volcano_MA.rar). GSA is a tool that uses semantic similarity and it is based on the IC (Information Content) proposed by Mazandu and Mulder [77]. Detailed analysis of gene expression and GSA analysis can be found in Supplementary Materials (Table S1). The RNA-seq data have been deposited at the NCBI’s Gene Expression Omnibus [78] and are accessible through GEO Series accession number GSE173614.

## 4. Conclusions

Here we investigated the virus-host interaction to resolve the phenomenon of improved lytic development of P1*vir* phage in a DksA-deficient *E. coli* host. We found that the P1*vir* virulence may be linked to the very high expression level of *ant1* and *kilA* genes. However, downregulated expression of genes directly responsible for triggering the lytic-only development in the *dksA^−^* host may result in less rapid and more gentle lysis. Moreover, modulation of phage lysozyme and the holin–antiholin system gene expression supports our hypothesis of gentle lysis as an explanation of the improved phage development in absence of DksA. We think that the longer the cell lysis is suppressed, the more virus particles can mature in a given time. In this context, the upregulation of morphogenesis and viral architecture genes supports the feasibility of other improvements in phage development.

We also found some interesting events taking place in the host cells upon infection. P1*vir* is taking control of the cellular protein, sugar, and lipid metabolism in both, the wild type and *dksA* mutant hosts. However, several genes involved in the sulfur metabolism were uniquely upregulated in the *dksA* mutant strain. It remains unclear if that is associated with obtaining new energy sources or with global reprogramming via H_2_S signaling functions. Generally, the hosts are reacting by activating their SOS response or upregulating the heat shock proteins. However, we also found downregulation of proteolysis which was unique for the *dksA^−^* strain.

We believe that this extensive and comprehensive study not only finds reasonable explanations for the improved P1*vir* development in the *dksA^−^* strain but also makes a great contribution to the field of P1 phage biology. However, we presume that our involvement in this vast story is quite not finished and we have not reached the bottom of *the Rabbit Hole*. 

## Data Availability

The data presented in this study are openly available in NCBI’s Gene Expression Omnibus; GEO accession number GSE173614.

## Supplementary Materials

The following are available online at https://www.mdpi.com/article/10.3390/ijms22116159/s1, Table S1: Gene Expression Data.xlsx; File archive: Suplementary_plots_Volcano_MA.rar

## Appendix A

**Table A1:** The most up- and downregulated host genes. The following genes of the wild type or *dksA* mutant hosts were found to be the most up- or downregulated hosts genes upon phage infection (regardless the time points). Double plus (++) represents host genes listed as the top ten most regulated at each time-point (the same as shown in Figure 3C or Figure 3D). One plus (+) indicates the host genes that were up- or downregulated, but their expression was lower than the top ten (therefore they were not shown in Figure 3). Minus (−) indicates that a gene was neither up- nor downregulated in the host. Arrows (**↓**↑) indicate the direction of regulation.

↓↑ Reg.	WT	*dksA*	Gene	Function
↓	++	++	*cpxP*	periplasmic adaptor protein
↓	++	++	*cspA*	RNA chaperone and antiterminator, cold-inducible
↓	++	++	*cspB*	Qin prophage; cold shock protein
↓	++	++	*cspG*	cold shock protein homolog
↓	++	++	*lpxP*	palmitoleoyl-acyl carrier protein
↓	++	++	*mdtI*	multidrug efflux system transporter
↓	++	++	*mdtJ*	multidrug efflux system transporter
↓	++	++	*nepI*	putative transporter
↓	++	++	*yncD*	putative iron outer membrane transporter
↓	++	+	*cspF*	Qin prophage, cold shock protein
↓	++	+	*dtpA*	dipeptide / tripeptide permease A
↓	++	−	*hisD*	bifunctional histidinal/histidinol dehydrogenase
↓	++	−	*hisG*	ATP phosphoribosyltransfer-ase
↓	++	−	*kdpA*	potassium translocating ATPase
↓	++	−	*yccA*	modu-lator of FtsH protease, inner membrane protein
↓	++	−	*ydjN*	putative transporter
↓	++	−	*ydjO*	uncharacterized protein
↓	++	−	*ygbE*	DUF3561 family inner membrane protein
↓	+	++	*bluF*	anti-repressor for YcgE
↓	+	++	*mcbR*	biofilm gene transcriptional regulator
↓	+	++	*ylaB*	put. membrane cyclic-diGMP phosphodiesterase
↓	−	++	*cyoA*	cyto-chrome o ubiquinol oxidase subunit II
↓	−	++	*pdhR*	pyruvate dehydrogenase complex repressor
↓	−	++	*rhlE*	ATP-dependent RNA helicase
↓	−	++	*xseA*	exonuclease VII
↓	−	++	*ydcX*	DUF2566 family protein
↓	−	++	*yjcB*	putative inner membrane protein
↑	−	++	*cdd*	cytidine/deoxycytidine deaminase
↑	++	+	*fxsA*	suppressor of F exclusion of phage T7
↑	++	+	*ibpA*	heat shock chaperone
↑	++	+	*ibpB*	heat shock chaperone
↑	++	−	*insI1*	IS30 transposase
↑	++	−	*ogrK*	orphan Ogr protein, positive regulator of P2 growth
↑	++	++	*pgaA*	biofilm adhesin polysaccharide PGA secretin
↑	++	+	*pgaB*	outer membrane export lipoprotein
↑	++	+	*phoU*	negative regulator of PhoR/PhoB two-component regulator
↑	++	+	*pstA*	phosphate ABC transporter permease
↑	++	+	*pstS*	phosphate ABC transporter periplasmic binding protein
↑	++	+	*recA*	DNA recombination and repair protein
↑	++	−	*sra*	stationary-phase-induced ribosome-associated protein
↑	++	+	*uspG*	universal stress protein UP12
↑	++	+	*ybeL*	DUF1451 family protein
↑	++	−	*ycaC*	putative isochorismatase family hydrolase
↑	++	+	*ycjX*	DUF463 family protein, puatative P-loop NTPase
↑	++	++	*yjiY*	putative transporter
↑	++	+	*hdhA*	7-alpha-hydroxysteroid dehydrogenase, NAD-dependent
↑	−	++	*cysA*	sulfate/thiosulfate transporter subunit
↑	−	++	*cysC*	adenosine 5’-phosphosulfate kinase
↑	−	++	*cysD*	sulfate adenylyltransferase, subunit 2
↑	−	++	*cysH*	phosphoadenosine phosphosulfate reductase
↑	−	++	*cysI*	sulfite reductase, beta subunit, NAD(P)-binding
↑	−	++	*cysJ*	sulfite reductase, alpha subunit, flavoprotein
↑	−	++	*cysN*	sulfate adenylyltransferase, subunit 1
↑	−	++	*cysW*	sulfate/thiosulfate ABC transporter permease
↑	−	++	*fruA*	fused fructose-specific PTS enzymes
↑	−	++	*gatY*	D-tagatose 1,6-bisphosphate aldolase 2, catalytic subunit
↑	−	++	*gatZ*	D-tagatose 1,6-bisphosphate aldolase 2, subunit
↑	−	++	*gntP*	fructuronate transporter
↑	−	++	*malP*	maltodextrin phosphorylase
↑	−	++	*udp*	uridine phosphorylase
↑	−	++	*yeeE*	UPF0394 family inner membrane protein
↑	−	++	*yhaO*	putative transporter
